# Preserved Muscle Strength Despite Muscle Mass Loss After Bariatric Metabolic Surgery: a Systematic Review and Meta-analysis

**DOI:** 10.1007/s11695-023-06796-9

**Published:** 2023-09-20

**Authors:** Han Na Jung, Seon-Ok Kim, Chang Hee Jung, Woo Je Lee, Myung Jin Kim, Yun Kyung Cho

**Affiliations:** 1 Division of Endocrinology and Metabolism, Department of Internal Medicine, Hallym University Sacred Heart Hospital, 22, Gwanpyeong-Ro 170Beon-Gil, Dongan-Gu, Anyang-Si, Gyeonggi-Do 14068 Republic of Korea; 2 Department of Biostatistics and Clinical Epidemiology, Asan Medical Center, University of Ulsan College of Medicine, 88, Olympic-Ro 43-Gil, Songpa-Gu, Seoul, 05505 Republic of Korea; 3 Asan Diabetes Center, Asan Medical Center, 88, Olympic-Ro 43-Gil, Songpa-Gu, Seoul, 05505 Republic of Korea; 4 Department of Internal Medicine, Asan Medical Center, University of Ulsan College of Medicine, 88, Olympic-Ro 43-Gil, Songpa-Gu, Seoul, 05505 Republic of Korea

**Keywords:** Bariatric surgery, Obesity, Muscle strength

## Abstract

**Background:**

Contrary to the previously known concept of muscle mass decrease following bariatric metabolic surgery, changes in muscle strength have been poorly investigated in systematic reviews. In this meta-analysis, we evaluated changes in handgrip strength (HGS) and lean mass (LM) after undergoing bariatric metabolic surgery.

**Methods:**

A systematic literature review using the PubMed, Embase, and Cochrane Library databases was conducted in November 2022. Longitudinal studies reporting HGS change after bariatric metabolic surgery were eligible. Pooled estimates for changes in HGS, body mass index (BMI), LM, and fat mass (FM) were calculated. Changes from baseline to the point closest to 6 months postoperatively were analyzed in trials with multiple follow-up examinations. The risk of bias was assessed using the Joanna Briggs Institute critical appraisal checklist.

**Results:**

Three randomized controlled trials and seven prospective cohort studies involving 301 patients were included. Follow-up evaluations were conducted 6 months postoperatively in all trials except for two, whose follow-up visits were at 18 weeks and 12 months, respectively. Pooled analysis showed reduced BMI (− 10.8 kg/m^2^; 95% confidence interval: − 11.6 to − 9.9 kg/m^2^), LM (− 7.4 kg; − 9.3 to − 5.4 kg), and FM (− 22.3 kg; − 25.1 to − 19.6 kg) after bariatric metabolic surgery, whereas the change in HGS was not statistically significant (− 0.46 kg; − 1.76 to 0.84 kg).

**Conclusion:**

Despite the decreased body composition parameters, including muscle mass, strength was not impaired after bariatric metabolic surgery; this indicates that bariatric metabolic surgery is an effective weight management intervention that does not compromise strength.

**Graphical Abstract:**

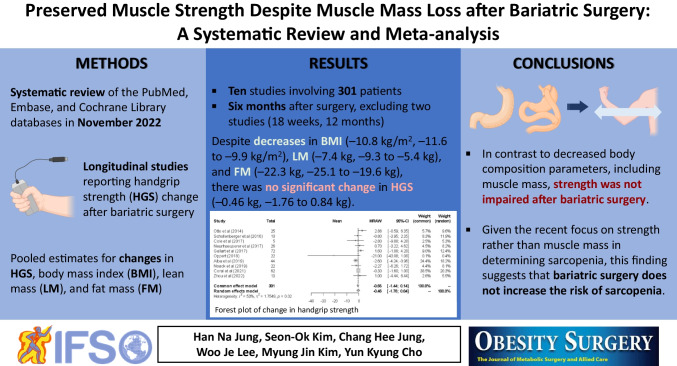

**Supplementary Information:**

The online version contains supplementary material available at 10.1007/s11695-023-06796-9.

## Introduction

Bariatric metabolic surgery is a well-established weight management method with robust evidence of safety and efficacy for profound reduction in weight and associated medical problems [1, 2]. However, muscle mass decreases after bariatric metabolic surgery, have raised concern regarding the increased risk of sarcopenia [3, 4]. Significant postoperative loss of muscle mass, as assessed by bioelectrical impedance analysis (BIA), dual-energy X-ray absorptiometry (DXA), magnetic resonance imaging (MRI), or plethysmography, is apparent within 1 month, and over half of the annual loss occurs within 3 months of surgery [3, 4]. Therefore, efforts have been made to measure muscle mass perioperatively to estimate the likelihood of sarcopenia [5].

The 2010 European Working Group on Sarcopenia in Older People (EWGSOP) definition of sarcopenia required an evidence of low muscle mass [6]. However, clinical implications of strength versus muscle mass are emerging [7, 8]. In a 17-country prospective analysis, lower handgrip strength (HGS) was associated with increased cardiovascular and non-cardiovascular mortality [7]. Similarly, the health, aging, and body composition study of older participants found that knee extension and HGS were better predictors of mortality than muscle size, as measured by computed tomography or DXA. Accordingly, the 2018 update of the European consensus for the diagnosis of sarcopenia prioritizes strength over muscle mass [9]. Specifically, HGS is the preferred screening test in sarcopenia diagnostic guidelines due to its convenience, cost-effectiveness, and reliability [9–11].

Despite the recognized importance of strength, a few studies have examined the effect of bariatric metabolic surgery on strength. Only one previous meta-analysis on physical function reported changes in strength after bariatric metabolic surgery [12]. However, it included literature published through 2015, and only five trials were included in the pooled analysis of strength [12]. Furthermore, the results were limited as strength data from different measurement methods and body parts were combined to calculate a pooled estimate [12]. Thus, we investigated changes in HGS after bariatric metabolic surgery and changes in body composition, including lean mass (LM) and fat mass (FM).

## Methods

### Search Strategy and Study Selection

The literature search approach was based on the population, intervention, comparison, and outcome protocol. This systematic review examined the adherence to the Preferred Reporting Items for Systematic Reviews and Meta-Analyses [13]. The PubMed, Embase, and Cochrane Library databases were searched for studies written in English and published between their inception to November 2, 2022. The search terms are listed in Supplementary Table 1. In addition, a manual search was conducted on the references cited in the identified articles.

Eligible articles were longitudinal studies on patients who underwent bariatric metabolic surgery that reported changes in HGS or provided baseline and postoperative HGS data. Two researchers (HNJ and YKC) independently examined the identified articles to determine whether they met the inclusion criteria. Differences in opinions regarding eligibility were resolved through consensus.

### Data Extraction and Quality Assessment

The primary outcome was the difference in the HGS in kg before and after bariatric metabolic surgery. The secondary outcomes were differences in body mass index (BMI; kg/m^2^), LM (kg), and FM (kg). Two independent investigators used a standardized data extraction form to gather the following data: author name; year of publication; study location; study design; follow-up interval; number of patients; percentage of men; type of bariatric metabolic surgery; method of measuring HGS and body composition; and baseline data including age, BMI, HGS, LM, and FM. Moreover, information on the mean changes in HGS, BMI, LM, and FM after bariatric metabolic surgery was obtained, along with information on measures of variability such as standard deviation (SD). If a study revealed only baseline and postoperative values, the mean changes were computed by subtracting the average baseline value from the average postoperative value. The SD of the difference between the two values was calculated by assuming a correlation of 0.5 between them [14]. For trials with multiple follow-up examinations, the time point for postoperative data was set to be the closest to 6 months postoperatively. The reason for choosing a 6-month interval is that it represents the period of most pronounced loss of weight and muscle mass and is, therefore, the period during which muscle strength is most likely to be affected [3, 15]. In multi-arm trials, patients who underwent interventions other than bariatric metabolic surgery were excluded from the analysis.

Two researchers (HNJ and YKC) independently evaluated the methodological quality of individual studies according to the Joanna Briggs Institute critical appraisal checklist [16]. Discrepancies in the quality assessment were resolved through discussion.

### Statistical Analysis

Pooled estimates of the changes in HGS, BMI, LM, and FM after bariatric metabolic surgery were analyzed. Subgroup analyses of the primary outcome were performed based on the type of bariatric metabolic surgery, percentage of men, and hand side for HGS measurement. A random-effects model was conducted to combine the studies, and forest plots were used to depict the results. Heterogeneity among the included studies was examined using the *I*^2^ statistic and Cochrane *Q* test [17]. The degree of heterogeneity was considered low, moderate, or high according to *I*^2^ values of 25%, 50%, and 75%, respectively [17]. Evidence of heterogeneity between studies was defined as a Q statistic with a *P* value of < 0.1 [17]. Funnel plots were used to detect publication bias with asymmetry indicating the potential for publication bias. Statistical analyses were conducted using the R statistical software (R Foundation, version 4.2.0).

## Results

### Study Selection

**Fig. 1:**
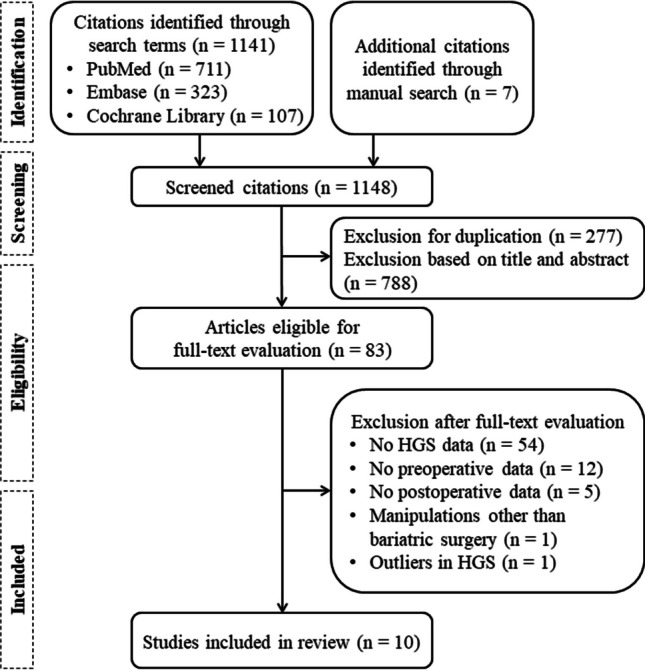
Study selection flowchart. HGS, handgrip strength

Of the 1148 citations obtained during the literature search, 10 studies involving patients who underwent bariatric metabolic surgery were eligible for inclusion in the meta-analysis (Fig. 1.) [18–27]. Most excluded studies after a full-text review either had no data on HGS or only provided baseline or postoperative data, making it impossible to calculate the changes. A study by Smelt et al. was excluded as it required all patients to receive a specific dose of vitamin D, which could have confounded the effect of bariatric metabolic surgery on outcomes [28]. Moreover, data from a study by Stegen et al. were excluded owing to the exceptionally high HGS values compared with those in the other included studies (baseline HGS, 95.9 kg vs. 25.7–36 kg), despite using the same measurement tools and units [29].

Of the 10 included studies, seven were single-arm prospective cohort studies [18, 20–22, 24, 26, 27], and three were randomized controlled trials [19, 23, 25] (Table 1). Schollenberger et al. divided patients into either a postsurgical protein supplementation group or a control group that received an isocaloric placebo [19]. Oppert et al. randomized patients into three categories: exercise plus protein supplementation, protein supplementation only, and control [23]. A study by Noack-Segovia et al. divided patients into exercise and control groups [25]. We included only control groups from randomized controlled trials to align with patients from the remaining cohort studies who received usual care after undergoing bariatric metabolic surgery; this resulted in 301 patients included in the pooled analysis.

**Table 1:** Summary of the studies included in the analysis

Author, year [ref]	Country	Study design	F/U interval	N	Men, %	Surgery type	Measurement method		Baseline data				
							HGS (dynamometer)	Body composition	Age, years	BMI, kg/m^2^	HGS, kg	LM, kg	FM, kg
Otto, 2014 [18]	Germany	PCS	18 W	25	36.0	SG, RYGB	Average of 3 trials with each hand (dominant hand data was used)	BIA	40.4 ± 10.8	47.4 ± 6.3	31.2 ± 7.0	71.5 ± 13.8	64.6 ± 15.8
Schollenberger, 2016 [19]	Germany	RCT	6 M	10	20.0	SG, RYGB	NA	BIA	47.0 ± 11.9	49.0 ± 5.1	30.0 ± 4.9^a^	68.9 ± 13.4	68.2 ± 12.0
Cole, 2017 [20]	USA	PCS	6 M	5	0.0	RYGB	Average of 4 trials, 2 from each hand	DXA	47.2 ± 10.9	48.8 ± 9.7	31.9 ± 6.1	63.5 ± 13.2	75.4 ± 22.6
Neunhaeuserer, 2017 [21]	Italy	PCS	6 M	26	30.8	SG	Average of 3 trials with each hand (right hand data was used)	NA	48.2 ± 9.0	45.2 ± 5.8	32.0 ± 9.3	NA	NA
Gallart-Aragón, 2017 [22]	Spain	PCS	6 M	72	34.7	SG	Average of 3 trials with each hand (dominant hand data was used)	NA	45.4 ± 9.4	46.5 ± 5.6	31.1 ± 11.5	NA	NA
Oppert, 2018 [23]	France	RCT	6 M	22	0.0	RYGB	Maximum of 5 trials with each hand	DXA	43.9 ± 10.7	43.6 ± 6.2	30.6 ± 5.7	55.6 ± 8.4	58.7 ± 11.8
Alba, 2019 [24]	USA	PCS	12 M	44	21.3	RYGB	Maximum with dominant hand	DXA	45 ± 12	44 ± 7	NA^b^	62 ± 12	56 ± 12
Noack-Segovia, 2019 [25]	Chile	RCT	6 M	22	25.6^c^	NA	Maximum of 3 trials with each hand	BIA	33 ± 6.9^c^	36.7 ± 3.3	34.2 ± 8.9	61.5 ± 13.5	41.0 ± 6.4
Coral, 2021 [26]	Brazil	PCS	6 M	62	16.1	SG, RYGB	Median of 3 trials	BIA	38.4 ± 10.8	42.2 ± 0.7	25.7 ± 1.2	32.5 ± 0.8	55.8 ± 1.3
Zhou, 2022 [27]	Belgium	PCS	6 M	13	46.2	SG, RYGB	Using minimum 3 and maximum 1 of 5 trials	DXA	49 ± 14	39.5 ± 3.5	36 ± 9	56 ± 10	53 ± 9

Data are presented as mean (standard deviation) unless otherwise specified

^a^The data are expressed in pounds and converted using a rate of 1 lb = 0.454 kg

^b^Only the change in HGS is presented, not the baseline data

^c^Only the combined sex ratio and mean age of the control (bariatric metabolic surgery only) and intervention (bariatric metabolic surgery plus physical exercise) groups are reported

*F/U*, Follow-up; *N*, number of patients; *HGS*, handgrip strength; *BMI*, body mass index; *LM*, lean mass; *FM*, fat mass; *PCS*, prospective cohort study; *W*, weeks; *SG*, sleeve gastrectomy; *RYGB*, Roux-en-Y gastric bypass; *BIA*, bioelectrical impedance analysis; *RCT*, randomized controlled trial; *M*, months; *NA*, not available; *DXA*, dual-energy X-ray absorptiometry

### Baseline Characteristics and Risk of Bias

Table 1 provides an overview of the outlines of the selected studies. The studies were conducted in European countries [18, 19, 21–23, 27], the USA [20, 24], and South America [25, 26] (Table 1). Except for the studies by Otto et al. and Alba et al., which carried out follow-up evaluations at 18 weeks and 12 months, respectively [18, 24], all other studies carried out follow-up evaluations at 6 months postoperatively [19–23, 25–27] (Table 1). Women were predominant in all studies [18–27], with only women included in the studies by Cole et al. and Oppert et al. [20, 23] (Table 1). The types of surgery were sleeve gastrectomy (SG) and Roux-en-Y gastric bypass (RYGB) [18–27], although Noack-Segovia et al. did not provide details on the specific surgery type [25] (Table 1).

The patients’ average age and preoperative BMI were 30–50 years and 36.7–49.0 kg/m^2^, respectively [18–27] (Table 1). The studies used varying measurement methods for HGS, with definitions based on mean, median, or maximum values, and measurements taken from the dominant hand, right hand, or both hands [18–27] (Table 1). All studies except those by Neunhaeuserer et al. and Gallart-Aragón et al. reported body composition data obtained using BIA or DXA, with the muscle index represented by LM [18–20, 23–27] (Table 1).

A comprehensive assessment of study quality is presented in Supplementary Table 2, which reveals that all the 10 studies showed a low to moderate risk of bias.

### Changes in HGS, BMI, and Body Composition

**Fig. 2:**
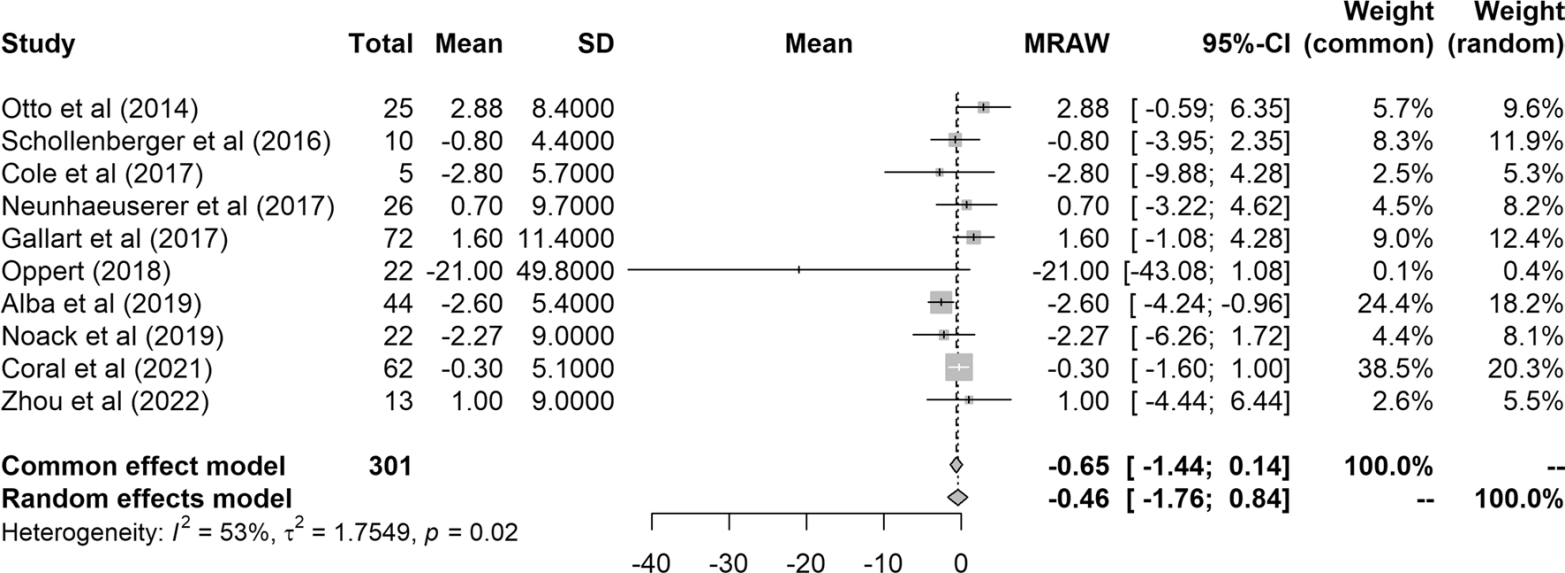
Forest plot of a meta-analysis of change in handgrip strength (kg) after bariatric metabolic surgery. The horizontal lines flanking the squares indicate the 95% confidence intervals. The diamonds depict the combined estimates

Postoperative HGS did not show a significant change from baseline in any of the studies, except for that by Alba et al., who reported a postoperative decrease in HGS (Fig. 2). The pooled analysis of HGS showed no significant change after bariatric metabolic surgery (mean difference [MD]: − 0.5 kg; 95% confidence interval [CI]: − 1.8 to 0.8 kg).

**Fig. 3:**
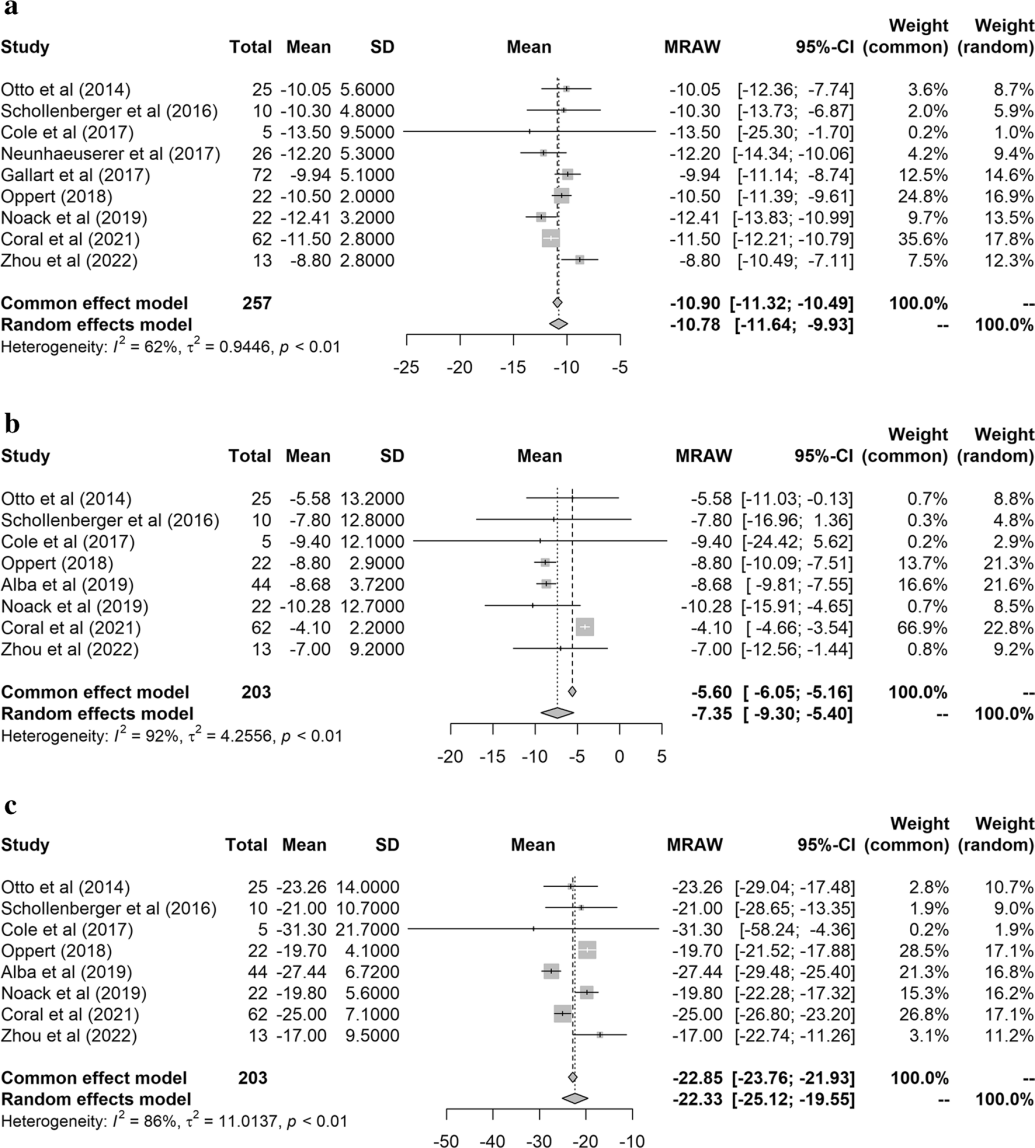
Forest plots of meta-analyses of changes in **a** body mass index (kg/m^2^), **b** lean mass (kg), and **c** fat mass (kg) after bariatric metabolic surgery. The horizontal lines flanking the squares indicate the 95% confidence intervals. The diamonds depict the combined estimates

Regarding the secondary outcomes, only studies providing data were included, with BMI analyzed in 257 patients from nine studies and LM and FM in 203 patients from eight studies (Fig. 3). Significant reductions in BMI, LM, and FM were observed in all studies except for those by Schollenberger et al. and Cole et al., where the decrease in LM was not statistically significant. In the pooled analysis, all BMI (MD: − 10.8 kg/m^2^; 95% CI: − 11.6 to − 9.9 kg/m^2^), LM (MD: − 7.4 kg; 95% CI: − 9.3 to − 5.4 kg), and FM (MD: − 22.3 kg; 95% CI: − 25.1 to − 19.6 kg) were significantly reduced after bariatric metabolic surgery.

The *I*^2^ statistic and Cochrane *Q* test indicated moderate heterogeneity between studies for HGS and BMI, but high heterogeneity for LM and FM. Considering the unusually low baseline LM observed in the study by Coral et al. (32.5 kg in their study vs. 55.6–71.5 kg in other studies) [26], the LM analysis was performed excluding the study by Coral et al., which significantly reduced heterogeneity (I2 decreased from 92 to 0%). This finding suggests that the high heterogeneity in LM can be attributed to the low baseline LM observed in the study by Coral et al. However, LM still significantly decreased from baseline in the pooled analysis, excluding the study by Coral et al. (MD: − 8.7 kg; 95% CI: − 9.4 to − 7.9 kg). Meanwhile, a subgroup analysis by continent for FM improved heterogeneity within the studies conducted in Europe and North America, with *I*^2^ values of 0% in both continents. The South American subgroup showed persistent high heterogeneity with an *I*^2^ value of 92%. However, further analysis was not feasible owing to the limited number (2) of the included studies. Overall, continents influence heterogeneity in the FM analysis, but significantly reduced FM was observed in all subgroups by continent (MD: − 19.8 kg [95% CI: − 21.3 to − 18.3 kg] for Europe; MD: − 27.5 kg [− 29.5 to − 25.5 kg] for North America; MD: − 22.5 kg [− 27.6 to − 17.4 kg] for South America). Funnel plots revealed a low possibility of publication bias in the meta-analysis (Figs. S1 and S2).

A subgroup analysis based on the type of bariatric metabolic surgery on the change in HGS revealed no significant change in the pooled analysis of the three studies with SG (MD: 0.2 kg; 95% CI: − 1.4 to 1.8 kg) and low heterogeneity was noted (Fig. S3). Moreover, a meta-analysis of four studies with RYGB reported no significant difference in postoperative HGS compared with that at baseline (MD: − 1.7 kg; 95% CI: − 4.1 to 0.7 kg), although the degree of heterogeneity was moderate to high. In addition, a subgroup analysis was conducted by stratifying studies based on the percentage of men below or above 30% (Fig. S4). In the pooled analysis of trials with < 30% of men, HGS was reduced by 1.5 kg (95% CI: − 2.9 to − 0.2 kg), whereas in trials with > 30% of men, HGS did not change significantly, with improved heterogeneity in both subgroups. Stratification by hand side revealed no significant changes in HGS across all subgroups (Fig. S5).

## Discussion

Despite BMI, LM, and FM decreases after bariatric metabolic surgery, HGS was unchanged in a meta-analysis of longitudinal data. The pooled analysis for each type of surgery, SG and RYGB, indicated no significant changes in HGS with either procedure. This study not only summarizes the current knowledge on perioperative changes in strength, but also provides evidence that bariatric metabolic surgery is an effective strategy for weight loss without compromising strength.

Two of the studies included in the meta-analysis reported postoperative changes in relative HGS [24, 27]. Alba et al. showed a significant improvement in the ratio of HGS to both BMI and appendicular LM, with percentage changes of 31.9% and 9.2%, respectively, 12 months after undergoing RYGB [24]. Zhou et al. reported that HGS divided by arm LM was significantly increased by 2.3 at 6 months after undergoing SG or RYGB [27]. The clinical significance of relative HGS was validated in several studies [30–32]. An analysis of data from the Korea National Health and Nutrition Examination Survey reported a negative association between higher quartiles of the HGS-to-BMI ratio and the risk of metabolic syndrome [30]. Cross-sectional population-based studies in Taiwan and China revealed an even stronger association between BMI-normalized HGS and healthy cardiometabolic parameters than with absolute HGS [31, 32]. A pooled analysis of the changes in relative HGS after bariatric metabolic surgery was not possible in the present study because of the small number of available studies. However, the relative HGS is expected to increase postoperatively, as most of the included studies showed a significant decrease in BMI and LM while maintaining HGS, which further supports the safety of bariatric metabolic surgery.

All studies in the meta-analysis revealed a nonsignificant change in HGS at the follow-up period [18–23, 25–27], whereas only Alba et al. reported a decrease at 6 and 12 months after bariatric metabolic surgery [24]. Nevertheless, relative HGS, presented as HGS divided by BMI or appendicular LM, was significantly enhanced in the study by Alba et al. [24] Furthermore, despite significant reductions in weight and LM during the 6- to 12-month interval, an upward trend was observed in absolute HGS and a significant increase in relative HGS during the same period [24]. Therefore, the study by Alba et al. reinforces the idea that changes in HGS after bariatric metabolic surgery are distinct from those in LM and other body composition measures.

The main explanation for the preservation of muscle strength as opposed to the loss of muscle mass following bariatric metabolic surgery may be the improvement in muscle quality. Increased infiltration of intramuscular fat exacerbates muscle stiffness and inflammation, leading to muscle weakness [33–35]. In contrast, microscopic examination revealed amelioration of lipid accumulation in the abdominal muscle 1 year after undergoing RYGB [36]. Similarly, a secondary analysis of the Longitudinal Assessment of Bariatric Surgery-2 revealed that intermuscular adipose tissue measured using whole body MRI, a widely accepted marker of muscular fat, decreased by 50% 12 months after bariatric metabolic surgery [37]. Weight reduction increases muscle oxidative capacity via promoting muscle capillarization and succinate dehydrogenase function, although its correlation to bariatric metabolic surgery has not been determined [38]. The enhancement of muscle quality resulting from bariatric metabolic surgery may counterbalance the negative effects of decreased muscle mass on muscle function.

Concerns regarding sarcopenia have increased owing to the loss of muscle mass following bariatric metabolic surgery [3, 4]. A recent meta-analysis on the change in LM over time after bariatric metabolic surgery reported a loss of 4.5 kg in the first 3 months and 8.1 kg by 12 months [3]; these results are consistent with our findings of a 7.4-kg loss in LM between 18 weeks and 12 months postoperatively. Similarly, another systematic review reported that time-varying fat-free mass (FFM) decreased by 5.6, 6.6, and 8.3 kg at 3, 6, and 12 months, respectively, after bariatric metabolic surgery [4]. In contrast, physical function, as measured by validated techniques such as the 6-min walk distance, short physical performance battery, or timed up-and-go test, is significantly enhanced by bariatric metabolic surgery [39]. Improvements in physical function were observed as early as 3 months postoperatively for the 6-min walk distance and even earlier for components of the short physical performance battery [39], whereas 55% and 67% of the 1-year loss occurred within 3 months for LM and FFM, respectively [3, 4].

One possible reason for the discrepancy between muscle mass and physical function after bariatric metabolic surgery is an increase in strength. Functional capacity is more strongly related to muscle strength than muscle mass [40–42]. Among the diagnostic indicators of sarcopenia, including low LM, HGS, and gait speed, poor HGS was the only independent risk factor for recurrent falls among participants in the longitudinal aging study Amsterdam [40]. A pooled analysis of prospective studies also showed that low muscle strength, rather than low muscle mass, was significantly associated with impaired physical function [41, 42]. The latest EWGSOP guidelines recommend strength over muscle mass for parameter of sarcopenia as muscle weakness is a better predictor of adverse outcomes [9]. Based on this evidence, bariatric metabolic surgery does not increase the risk of sarcopenia since postoperative strength is maintained despite body composition changes. Contrary to the misconception that sarcopenia is a disease of old age, the prevalence of sarcopenia ranges from 8.8 to 35.7% in healthy young adults around the average age of patients in this meta-analysis [43]. Our results proved that bariatric metabolic surgery can be performed in young age groups without concerns about sarcopenia.

This study had several limitations. First, the included studies were heterogeneous. This may be due to differences in countries, follow-up intervals, sex ratios, methods used to measure HGS and body composition, and other preoperative characteristics. Therefore, pooled estimates were generated using a random-effects model and subgroup analyses were performed. In particular, the improved heterogeneity in the subgroup analysis for the proportion of men showed a sex influence on HGS change, which requires further investigation of sex-specific data. Second, meta-analyses of strength measurements other than HGS were not possible, although weight-bearing muscles such as the back, pelvis, and lower extremities may reveal distinct outcomes. HGS was the most commonly used assessment tool in our systematic review of research assessing strength after bariatric metabolic surgery. Alternative methods were used infrequently, making it difficult to conduct their pooled analysis. Moreover, HGS is a valid surrogate for strength in other body parts, and the EWGSOP diagnostic algorithm for sarcopenia recommends it as an initial work-up [9–11]. The HGS appears to be the most pragmatic and generalizable tool for quantifying muscle strength after undergoing bariatric metabolic surgery; however, additional research is required to determine the most reliable measure. Third, the results might have been influenced by the exclusion of certain data owing to insufficient information, such as the HGS value in the study by Stegen et al. [29], surgical types in the study by Noack-Segovia et al. [25], or unknown hand side for measuring HGS in some studies [19, 26, 27]. Fourth, the change in LM, which we used as a proxy for muscle mass, only partially reflected the change in muscle mass as LM comprises organ and water content as well as muscle [3]. Finally, the studies included in the analysis were limited to non-Hispanic white or Hispanic populations, compromising the generalizability to any ethnicity.

Despite these limitations, we demonstrated that HGS was preserved while BMI, LM, and FM were reduced after bariatric metabolic surgery, thus refuting traditional doubts about postoperative sarcopenia. As muscle strength is recommended as the primary test to diagnose sarcopenia, generalization of perioperative strength measurements should be considered. Specifically, HGS is affordable, accessible, and has the most cumulative evidence in patients who have undergone bariatric metabolic surgery. Future research on the long-term effects of bariatric metabolic surgery on strength, muscle quality, and metabolic benefits associated with strength maintenance would facilitate the standardized assessment of perioperative strength in clinical practice.

## Conclusion

Muscle strength, as represented by HGS, was unaffected by bariatric metabolic surgery, which lowered LM and other body composition indices. Nowadays, strength is considered more clinically significant than muscular mass; however, there is a lack of sufficient data on changes in strength following bariatric metabolic surgery. In this context, our results underscore the safety of bariatric metabolic surgery in preserving muscle function.

### Supplementary Information

Below is the link to the electronic supplementary material.

Supplementary file1 (DOC 970 KB)

## Data Availability

All data analyzed during this study are included in this published article.
