# Supplementary material for: Preserved Muscle Strength Despite Muscle Mass Loss After Bariatric Metabolic Surgery: a Systematic Review and Meta-analysis
Source: Obes Surg. 2023 Sep 20;33(11):3422–30. doi: 10.1007/s11695-023-06796-9 (PMC10602996; doi:10.1007/s11695-023-06796-9)
Supplement: Supplementary file 1 — Supplementary file1 (DOC 970 KB) [file 11695_2023_6796_MOESM1_ESM.doc]

**Supplementary Material**

**Contents**

Table S1. Search strategy

Table S2. Joanna Briggs Institute critical appraisal checklist for the included studies

Figure S1. Funnel plot of change in handgrip strength after bariatric metabolic surgery.

Figure S2. Funnel plots of changes in (a) body mass index, (b) lean mass, and (c) fat mass after bariatric metabolic surgery.

Figure S3. Forest plots of meta-analyses of changes in handgrip strength (kg) after (a) sleeve gastrectomy and (b) Roux-en-Y gastric bypass.

Figure S4. Forest plot of a meta-analysis of change in handgrip strength (kg) after bariatric metabolic surgery in studies with proportion of men (a) above and (b) below 30%.

Figure S5. Forest plot of a meta-analysis of change in handgrip strength (kg) after bariatric metabolic surgery by hand side.

PRISMA checklist

PICO protocol

Table S1. Search strategy

| **Data source** | **Search terms** |
| --- | --- |
| PubMed | #1 "Obesity/surgery"[Mesh]  #2 "Bariatric Surgery"[Mesh]  #3 (Bariatric*[TW] OR Obes*[TW] OR metabolic*[TW] OR weight-loss[TW]) AND (Surger*[TW] OR Surgical[TW] OR operation*[TW] OR Procedur*[TW] OR anastomos*[TW])  #4 "Obesity"[Mesh] OR Obes*[TW] OR Bariatric*[TW]  #5 "Stomach Stapling"[TW] OR "stomach bypass"[TW] OR "Gastric Bypass"[TW] OR gastrojejunostom*[TW] OR "Gastroileal Bypass"[TW] OR "ileojejunal bypass"[TW] OR "intestinal bypass"[TW] OR "Roux-en-Y"[TW] OR Gastrojejunostom*[TW] OR Gastroplast*[TW] OR "Jejunoileal Bypass"[TW] OR "Jejunoileal Bypasses"[TW] OR Lipectom*[TW] OR Lipoabdominoplast*[TW] OR gastrectom*[TW] OR "vertical sleeve"[TW] OR "gastric band*"[TW] OR "Biliopancreatic Diversion"[Mesh] OR "Biliopancreatic Diversion*"[TW] OR "Bilio-Pancreatic Bypass"[TW] OR "duodenal switch"[TW]  #6 #4 AND #5  #7 **#1 OR #2 OR #3 OR #6**  #8 "Muscle Strength"[Mesh] OR "Hand Strength"[Mesh]  #9 strength[TW] AND (muscl*[TW] OR hand[TW] OR hands[TW] OR handgrip[TW] OR "lower limb*"[TW] OR dynamic[TW] OR quadriceps[TW] OR hamstring*[TW])  #10 "muscle contraction*"[TW] OR "muscular contraction*"[TW] OR "muscle force"[TW] OR "muscular force"[TW] OR "muscle power"[TW] OR "muscular power"[TW]  #11 "physical function"[TW]  #12 **#8 OR #9 OR #10 OR #11**  #13 **#7 AND #12**  #14 #13 NOT (animals[Mesh:noexp] NOT (animals[Mesh:noexp] AND humans[Mesh]))  #15 #14 NOT (Autobiography[ptyp] OR Bibliography[ptyp] OR Biography[ptyp] OR pubmed books[filter] OR Comment[ptyp] OR Dataset[ptyp] OR Dictionary[ptyp] OR Editorial[ptyp] OR Electronic Supplementary Materials[ptyp] OR Interview[ptyp] OR Legislation[ptyp] OR News[ptyp] OR Newspaper Article[ptyp] OR Retracted Publication[sb] OR Retraction of Publication[sb] OR Technical Report[ptyp] OR Letter[ptyp] OR "Case Reports" [Ptyp] OR case[TW])  #16 **#14 AND English[lang]** |
| Embase | #1 ''obesity'/exp/dm_su  #2 ''bariatric surgery'/exp  #3 ((Bariatric* OR Obes* OR metabolic* OR weight-loss) NEAR/6 (Surger* OR Surgical OR operation* OR Procedur* OR anastomos*)):ab,ti,kw #4 ''obesity'/exp OR (Obes* OR Bariatric*):ab,ti,kw  #5 '('Stomach Stapling' OR 'stomach bypass' OR 'Gastric Bypass' OR gastrojejunostom* OR 'Gastroileal Bypass' OR 'ileojejunal bypass' OR 'intestinal bypass' OR 'Roux-en-Y' OR Gastrojejunostom* OR Gastroplast* OR 'Jejunoileal Bypass' OR 'Jejunoileal Bypasses' OR Lipectom* OR Lipoabdominoplast* OR gastrectom* OR 'vertical sleeve' OR 'gastric band*'):ab,ti,kw  #6 #4 AND #5  #7 **#1 OR #2 OR #3 OR #6**  #8 ''muscle strength'/exp OR 'hand strength'/exp  #9 (strength NEAR/6 (muscl* OR hand OR hands OR handgrip OR 'lower limb*' OR dynamic OR quadriceps OR hamstring*)):ab,ti,kw  #10 '('muscle contraction*' OR 'muscular contraction*' OR 'muscle force' OR 'muscular force' OR 'muscle power' OR 'muscular power'):ab,ti,kw  #11 ''physical function':ab,ti,kw  #12 **#8 OR #9 OR #10 OR #11**  #13 **#7 AND #12**  #14 #13 NOT ('animal'/de NOT ('animal'/de AND 'human'/exp))  #15 #14 AND [english]/lim  #16 **#15 AND ([article]/lim OR [article in press]/lim OR [review]/lim)** |
| Cochrane | #1 [mh "Obesity"/SU]  #2 [mh "Bariatric Surgery"]  #3 ((Bariatric* OR Obes* OR metabolic* OR weight-loss) NEAR/6 (Surger* OR Surgical OR operation* OR Procedur* OR anastomos*)):ab,ti,kw  #4 [mh "Obesity"] OR (Obes* OR Bariatric*):ab,ti,kw  #5 '("Stomach Stapling" OR "stomach bypass" OR "Gastric Bypass" OR gastrojejunostom* OR "Gastroileal Bypass" OR "ileojejunal bypass" OR "intestinal bypass" OR "Roux-en-Y" OR Gastrojejunostom* OR Gastroplast* OR "Jejunoileal Bypass" OR "Jejunoileal Bypasses" OR Lipectom* OR Lipoabdominoplast* OR gastrectom* OR "vertical sleeve" OR "gastric band*"):ab,ti,kw  #6 #4 AND #5  #7 **#1 OR #2 OR #3 OR #6**  #8 [mh "Muscle Strength"] OR [mh "Hand Strength"]  #9 (strength NEAR/6 (muscl* OR hand OR hands OR handgrip OR "lower limb*" OR dynamic OR quadriceps OR hamstring*)):ab,ti,kw  #10 '("muscle contraction*" OR "muscular contraction*" OR "muscle force" OR "muscular force" OR "muscle power" OR "muscular power"):ab,ti,kw  #11 'physical function:ab,ti,kw  #12 **#8 OR #9 OR #10 OR #11**  #13 **#7 AND #12**  #14 **#13 in Reviews, Trials (incl. Pubmed, Embase)** |

**Table S2. Joanna Briggs Institute critical appraisal checklist for the included studies**

| **First Author (year)** | **Q1** | **Q2** | **Q3** | **Q4** | **Q5** | **Q6** | **Q7** | **Q8** | **Q9** | **Q10** | **Q11** | **Q12** | **Q13** | **%Y** | **Risk^a^** |
| --- | --- | --- | --- | --- | --- | --- | --- | --- | --- | --- | --- | --- | --- | --- | --- |
| Otto et al. (2014) | Y | Y | N | N | Y | Y | Y | Y | Y |  |  |  |  | 78 | low |
| Schollenberger et al. (2016) | Y | Y | Y | Y | Y | Y | Y | N | Y | Y | Y | Y | Y | 92 | low |
| Cole et al. (2017) | Y | Y | N | N | Y | Y | Y | Y | Y |  |  |  |  | 78 | low |
| Neunhaeuserer et al. (2017) | Y | Y | N | N | Y | Y | Y | N | Y |  |  |  |  | 67 | moderate |
| Gallart-Aragón et al. (2017) | Y | Y | N | N | Y | Y | Y | Y | Y |  |  |  |  | 78 | low |
| Oppert et al. (2018) | Y | N | Y | N | N | N | Y | Y | Y | Y | Y | Y | Y | 69 | moderate |
| Alba et al. (2019) | Y | Y | N | N | Y | Y | Y | Y | Y |  |  |  |  | 78 | low |
| Noack-Segovia et al. (2019) | Y | N | N | N | N | N | Y | Y | Y | Y | Y | Y | Y | 62 | moderate |
| Coral et al. (2021) | Y | Y | N | N | Y | Y | Y | Y | Y |  |  |  |  | 78 | low |
| Zhou et al. (2022) | Y | Y | Y | Y | Y | Y | Y | Y | Y |  |  |  |  | 100 | low |

The risk of potential bias was considered high when the percentage of "yes" responses was up to 49%, moderate when it ranged from 50 to 69%, and low when it exceeded 70%.

Q, question; Y, yes; N, no.

**Question codes for randomized controlled trials**

|  | **JBI Question** |
| --- | --- |
| Q1: | Was true randomization used for assignment of participants to treatment groups? |
| Q2: | Was allocation to treatment groups concealed? |
| Q3: | Were treatment groups similar at the baseline? |
| Q4: | Were participants blind to treatment assignment? |
| Q5: | Were those delivering treatment blind to treatment assignment? |
| Q6: | Were outcomes assessors blind to treatment assignment? |
| Q7: | Were treatment groups treated identically other than the intervention of interest? |
| Q8: | Was follow up complete and if not, were differences between groups in terms of their follow up adequately described and analyzed? |
| Q9: | Were participants analyzed in the groups to which they were randomized? |
| Q10: | Were outcomes measured in the same way for treatment groups? |
| Q11: | Were outcomes measured in a reliable way? |
| Q12: | Was appropriate statistical analysis used? |
| Q13: | Was the trial design appropriate, and any deviations from the standard RCT design (individual randomization, parallel groups) accounted for in the conduct and analysis of the trial? |

**Question codes for non-randomized experimental studies**

|  | **JBI Question** |
| --- | --- |
| Q1: | Is it clear in the study what is the ‘cause’ and what is the ‘effect’? |
| Q2: | Were the participants included in any comparisons similar? |
| Q3: | Were the participants included in any comparisons receiving similar treatment/care, other than the exposure or intervention of interest? |
| Q4: | Was there a control group? |
| Q5: | Were there multiple measurements of the outcome both pre and post the intervention/exposure? |
| Q6: | Was follow up complete and if not, were differences between groups in terms of their follow up adequately described and analyzed? |
| Q7: | Were the outcomes of participants included in any comparisons measured in the same way? |
| Q8: | Were outcomes measured in a reliable way? |
| Q9: | Was appropriate statistical analysis used? |


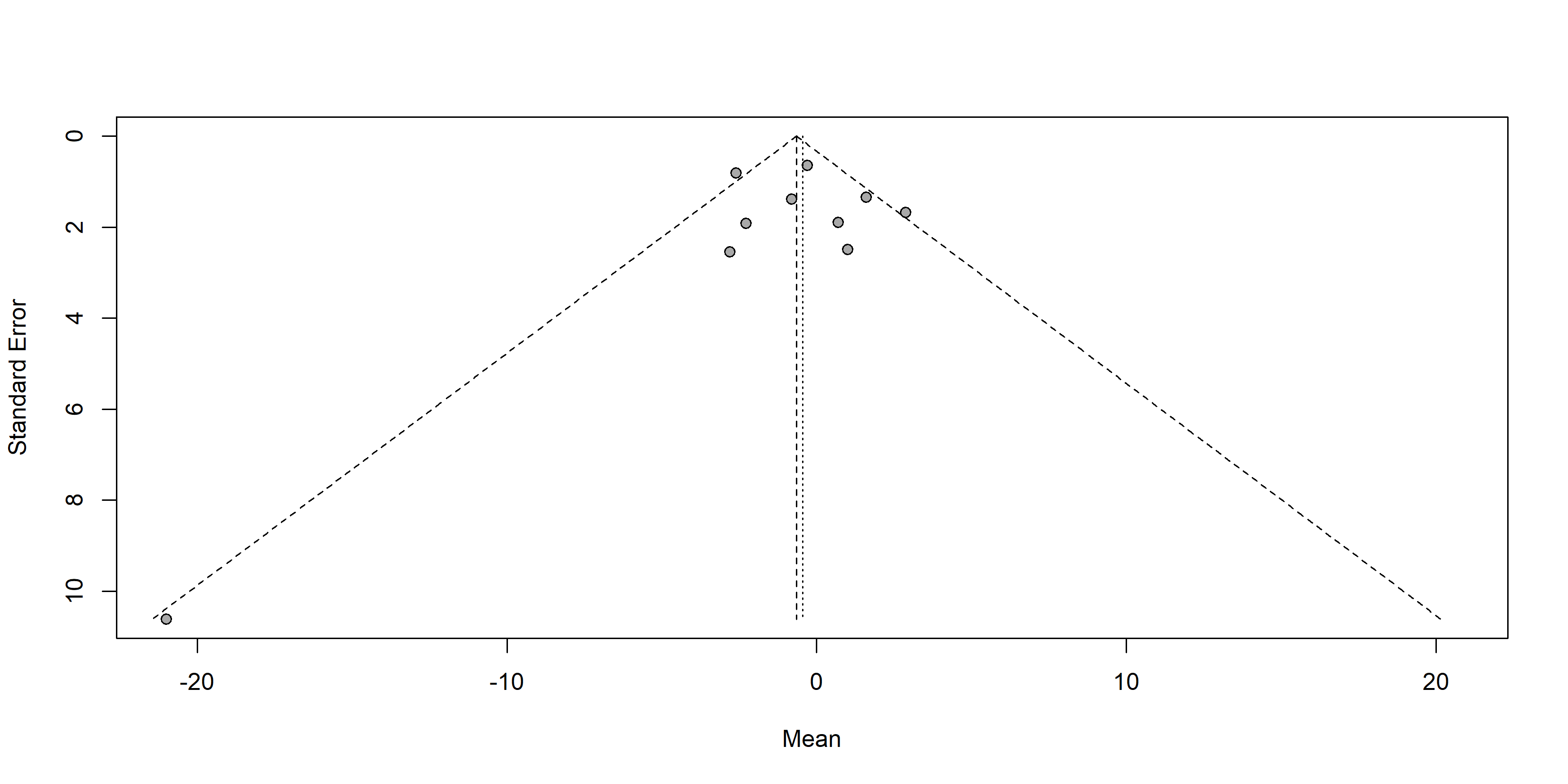


Figure S1. Funnel plot of change in handgrip strength after bariatric metabolic surgery.

(a) Body mass index


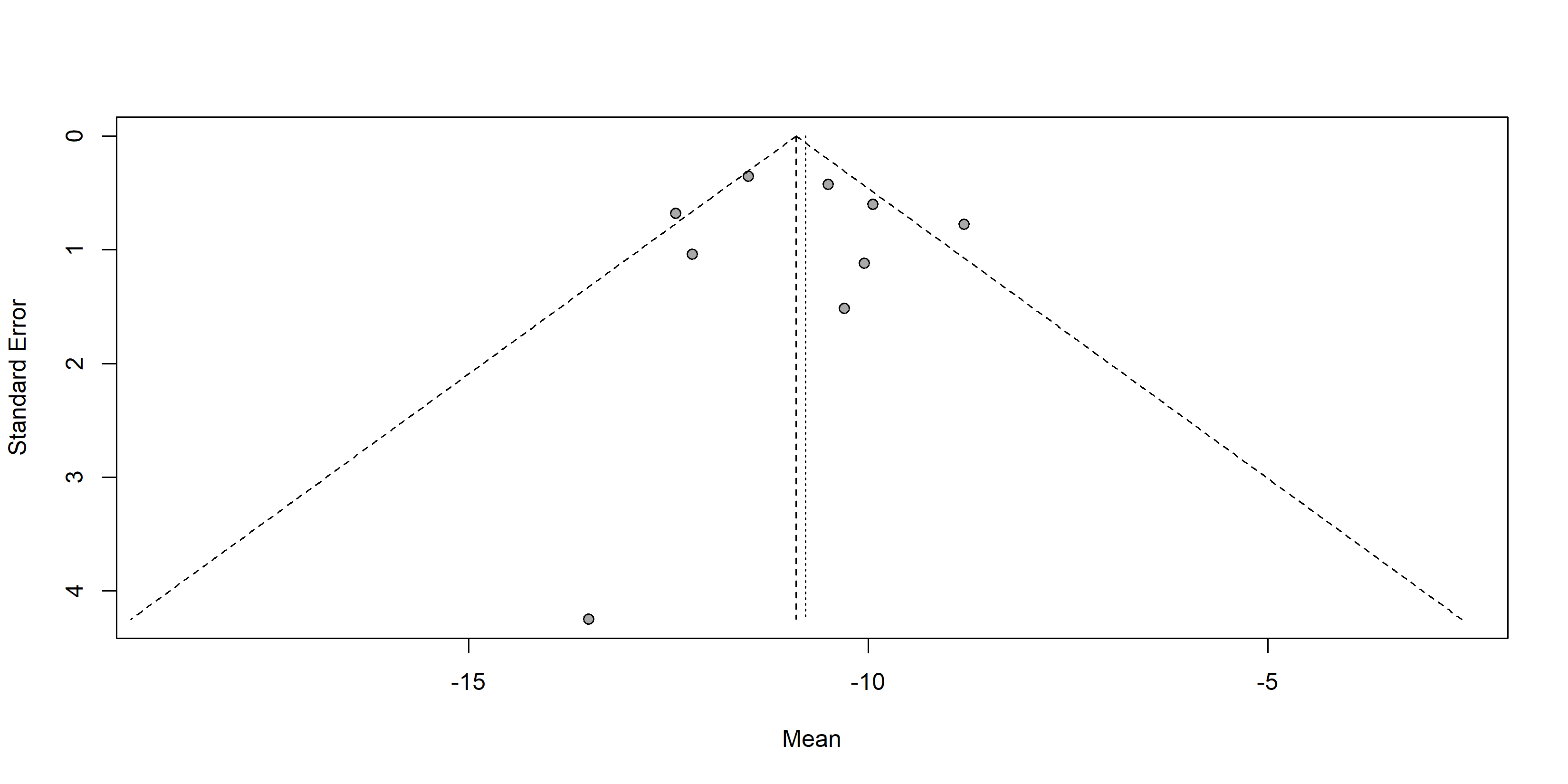


(b) Lean mass


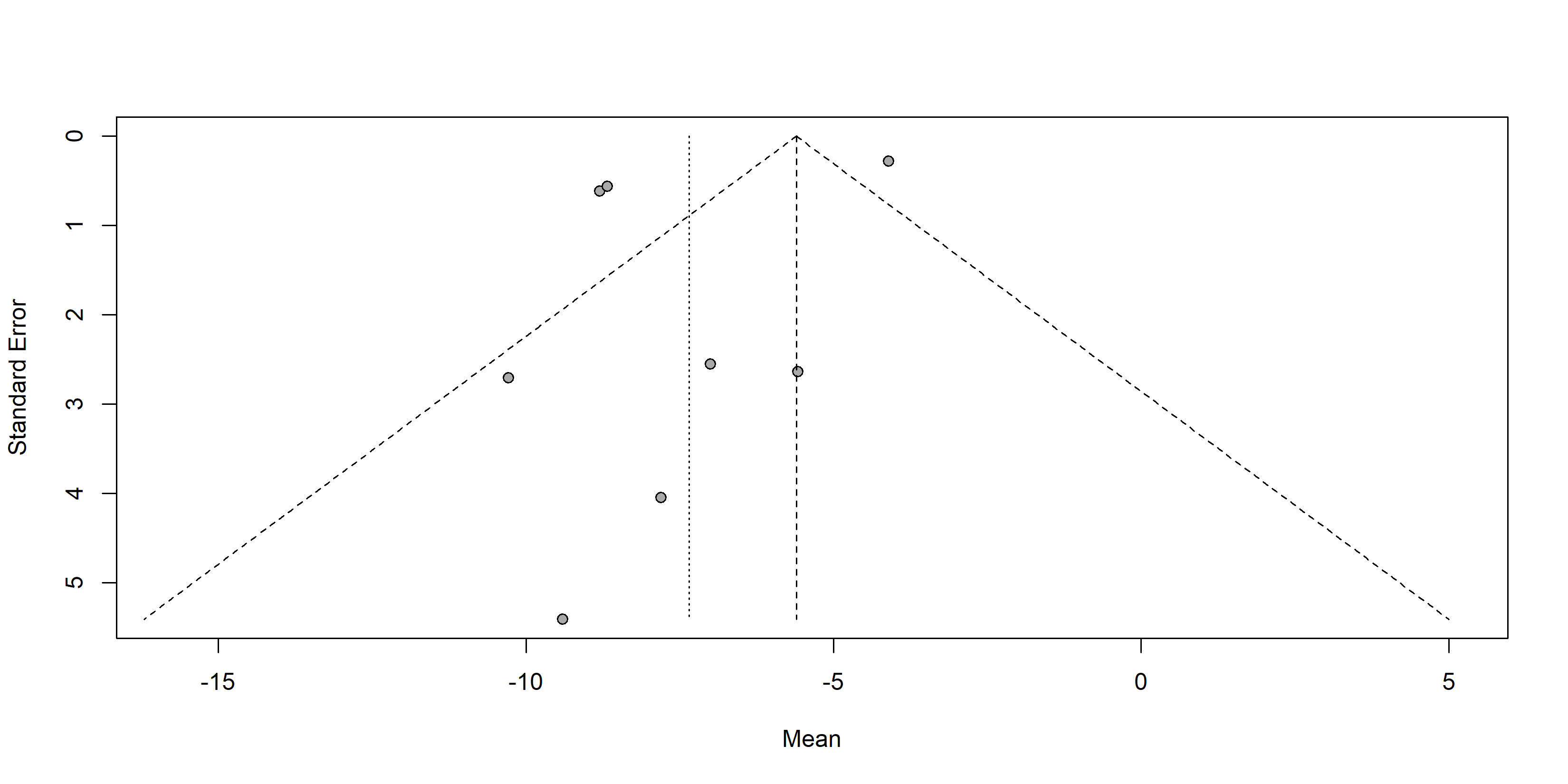


(c) Fat mass


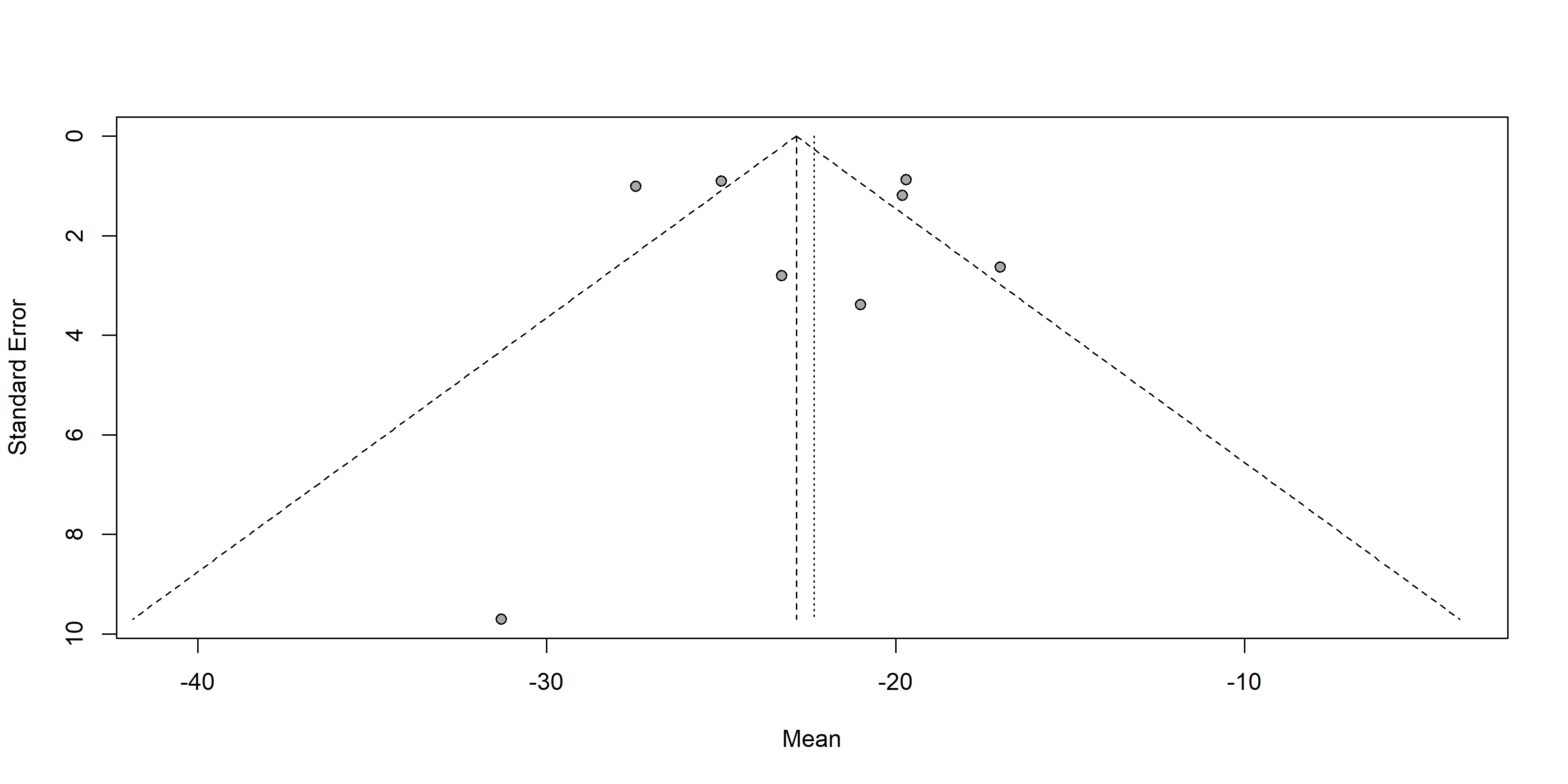


Figure S2. Funnel plots of changes in (a) body mass index, (b) lean mass, and (c) fat mass after bariatric metabolic surgery.

(a) Sleeve gastrectomy


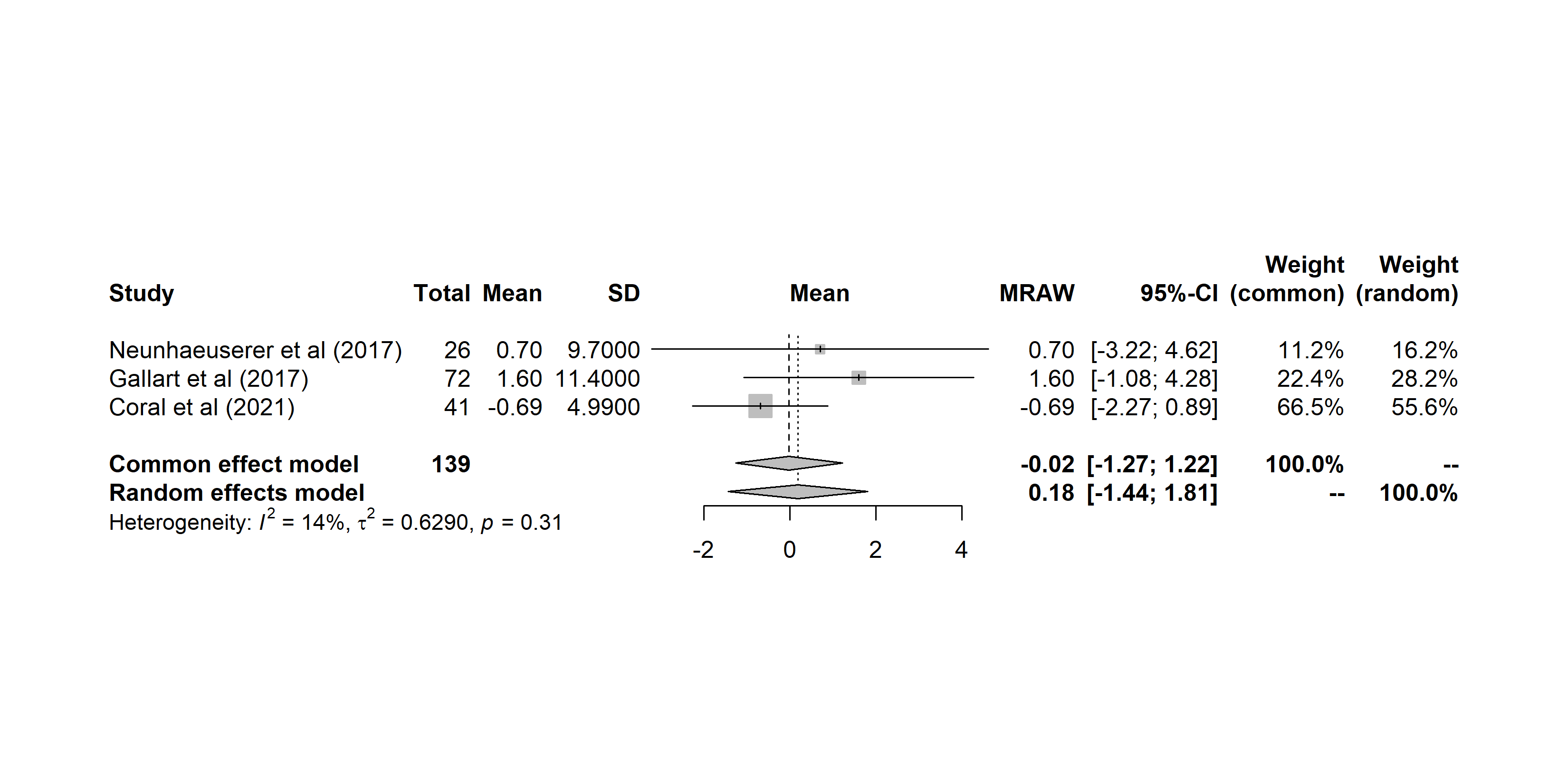


(b) Roux-en-Y gastric bypass


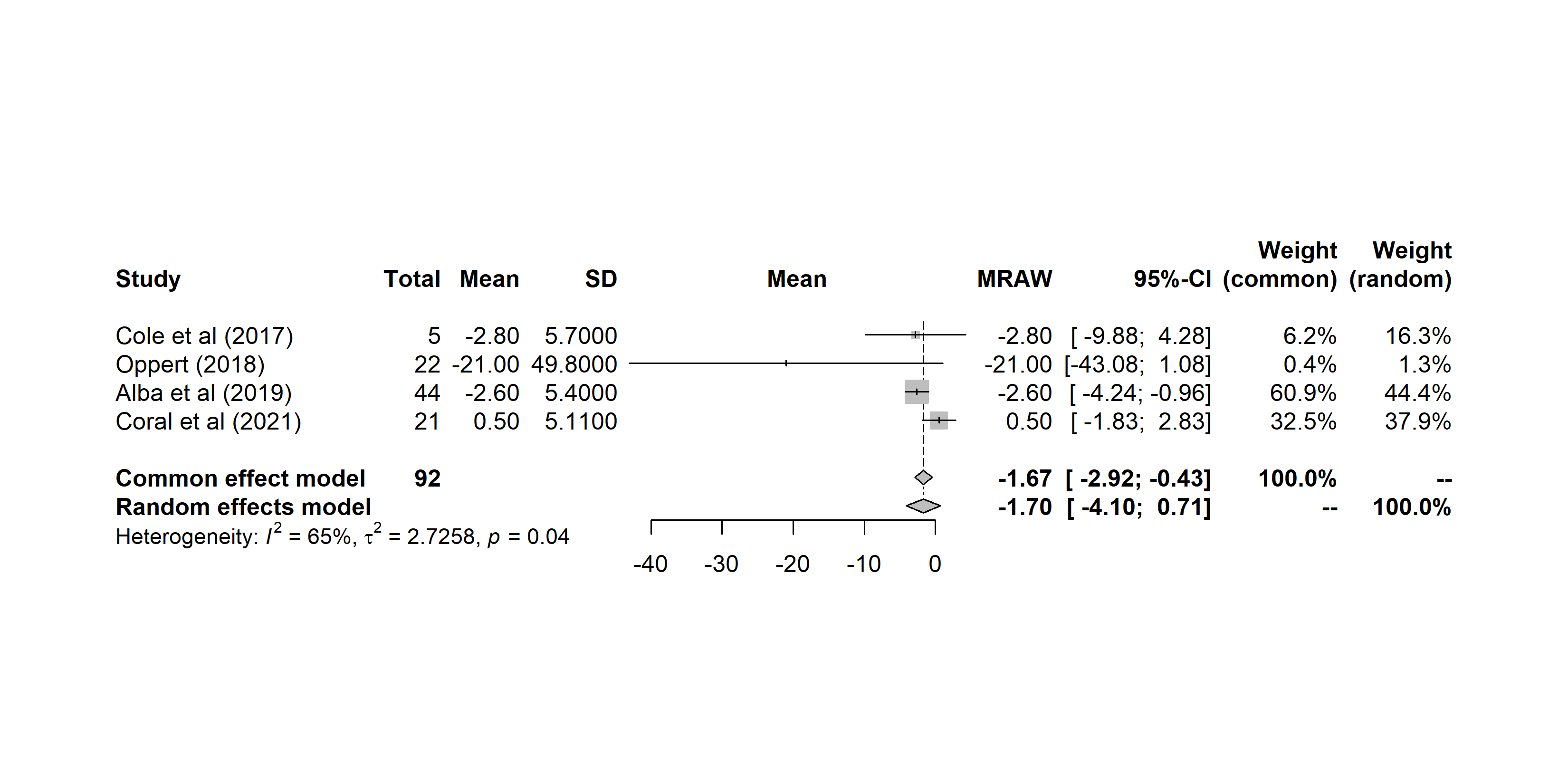


Figure S3. Forest plots of meta-analyses of changes in handgrip strength (kg) after (a) sleeve gastrectomy and (b) Roux-en-Y gastric bypass.

The horizontal lines flanking the squares indicate the 95% confidence intervals. The diamonds depict the combined estimates.


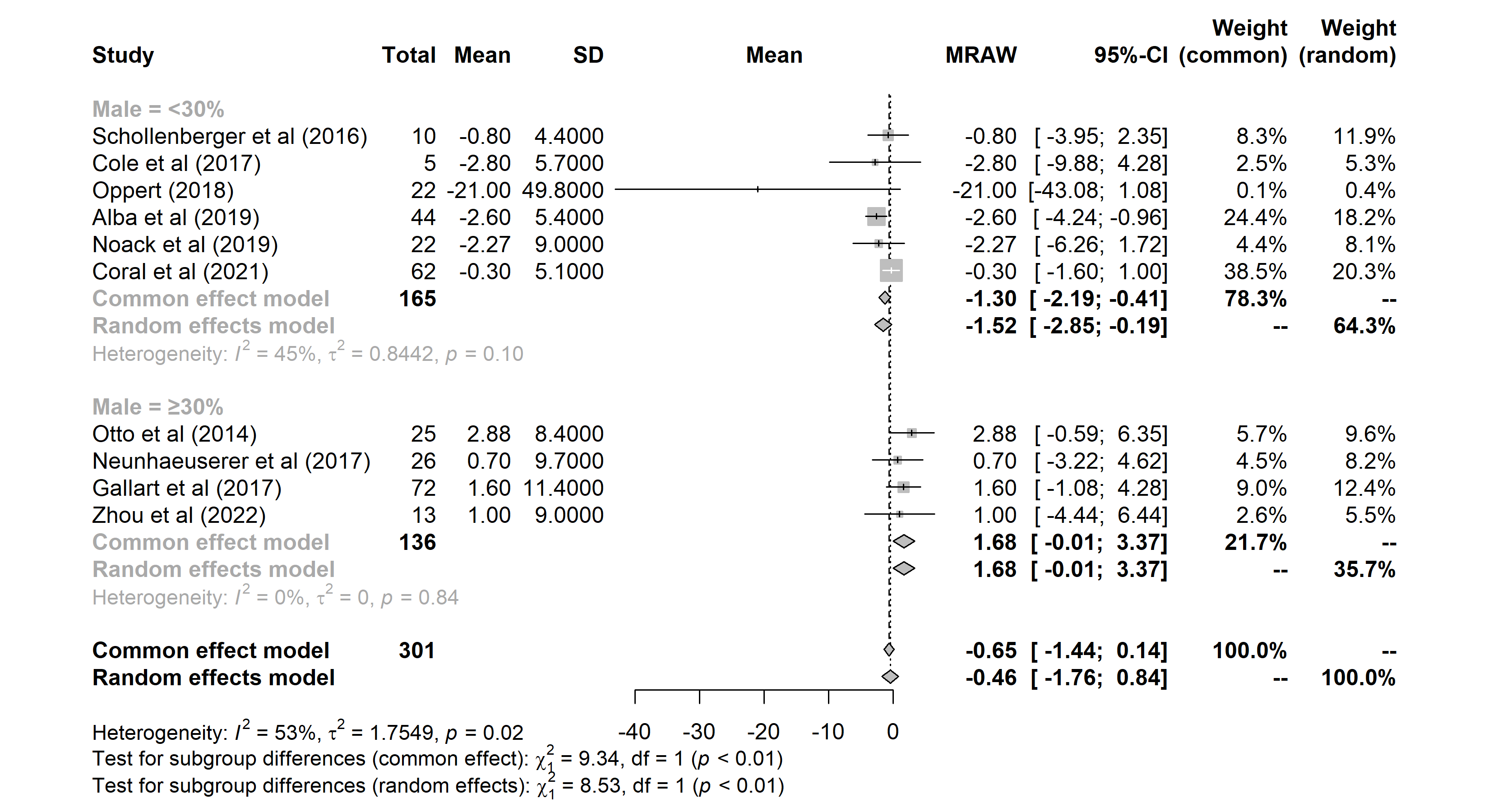


Figure S4. Forest plot of a meta-analysis of change in handgrip strength (kg) after bariatric metabolic surgery in studies with proportion of men (upper) below and (lower) above 30%.

The horizontal lines flanking the squares indicate the 95% confidence intervals. The diamonds depict the combined estimates.


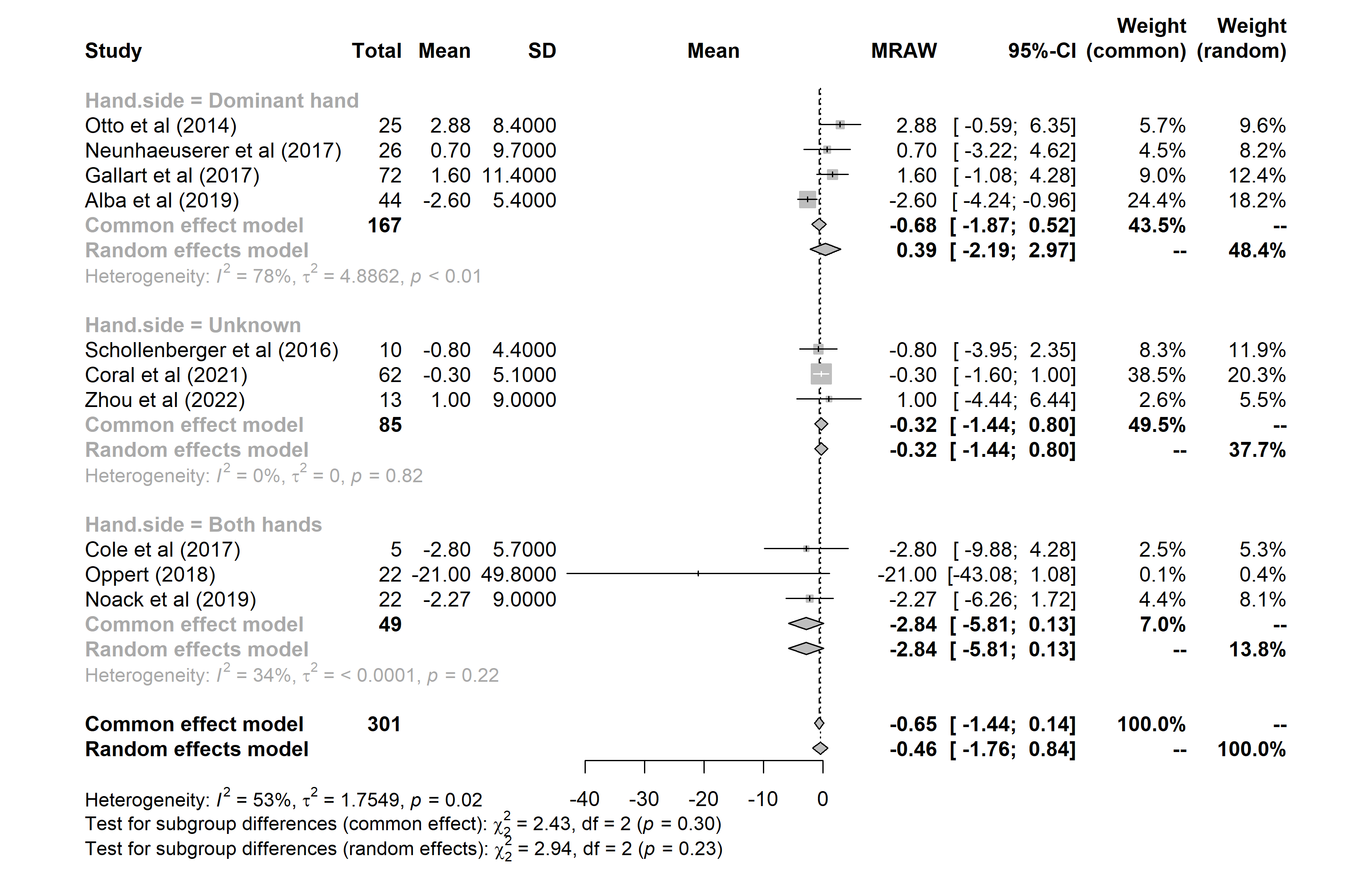


Figure S5. Forest plot of a meta-analysis of change in handgrip strength (kg) after bariatric metabolic surgery by hand side.

The horizontal lines flanking the squares indicate the 95% confidence intervals. The diamonds depict the combined estimates.

**PRISMA 2020 for abstracts chec**klist

| **Section and Topic** | **Item #** | **Checklist item** | **Reported (Yes/No)** |
| --- | --- | --- | --- |
| **TITLE** | | |  |
| Title | 1 | Identify the report as a systematic review. | Yes |
| **BACKGROUND** | | |  |
| Objectives | 2 | Provide an explicit statement of the main objective(s) or question(s) the review addresses. | Yes |
| **METHODS** | | |  |
| Eligibility criteria | 3 | Specify the inclusion and exclusion criteria for the review. | Yes |
| Information sources | 4 | Specify the information sources (e.g. databases, registers) used to identify studies and the date when each was last searched. | Yes |
| Risk of bias | 5 | Specify the methods used to assess risk of bias in the included studies. | Yes |
| Synthesis of results | 6 | Specify the methods used to present and synthesise results. | Yes |
| **RESULTS** | | |  |
| Included studies | 7 | Give the total number of included studies and participants and summarise relevant characteristics of studies. | Yes |
| Synthesis of results | 8 | Present results for main outcomes, preferably indicating the number of included studies and participants for each. If meta-analysis was done, report the summary estimate and confidence/credible interval. If comparing groups, indicate the direction of the effect (i.e. which group is favoured). | Yes |
| **DISCUSSION** | | |  |
| Limitations of evidence | 9 | Provide a brief summary of the limitations of the evidence included in the review (e.g. study risk of bias, inconsistency and imprecision). | No |
| Interpretation | 10 | Provide a general interpretation of the results and important implications. | Yes |
| **OTHER** | | |  |
| Funding | 11 | Specify the primary source of funding for the review. | N/A |
| Registration | 12 | Provide the register name and registration number. | N/A |

PRISMA, Preferred Reporting Items for Systematic Reviews and Meta-Analyses; N/A, not applicable.

**PRISMA 2020 checklist**

| **Section and Topic** | **Item #** | **Checklist item** | **Location where item is reported** |
| --- | --- | --- | --- |
| **TITLE** | | |  |
| Title | 1 | Identify the report as a systematic review. | Title page |
| **ABSTRACT** | | |  |
| Abstract | 2 | See the PRISMA 2020 for Abstracts checklist. | Page 1 |
| **INTRODUCTION** | | |  |
| Rationale | 3 | Describe the rationale for the review in the context of existing knowledge. | Page 3 |
| Objectives | 4 | Provide an explicit statement of the objective(s) or question(s) the review addresses. | Page 3 |
| **METHODS** | | |  |
| Eligibility criteria | 5 | Specify the inclusion and exclusion criteria for the review and how studies were grouped for the syntheses. | Page 4 |
| Information sources | 6 | Specify all databases, registers, websites, organisations, reference lists and other sources searched or consulted to identify studies. Specify the date when each source was last searched or consulted. | Page 4 |
| Search strategy | 7 | Present the full search strategies for all databases, registers and websites, including any filters and limits used. | Table S1 |
| Selection process | 8 | Specify the methods used to decide whether a study met the inclusion criteria of the review, including how many reviewers screened each record and each report retrieved, whether they worked independently, and if applicable, details of automation tools used in the process. | Page 4 |
| Data collection process | 9 | Specify the methods used to collect data from reports, including how many reviewers collected data from each report, whether they worked independently, any processes for obtaining or confirming data from study investigators, and if applicable, details of automation tools used in the process. | Page 4 |
| Data items | 10a | List and define all outcomes for which data were sought. Specify whether all results that were compatible with each outcome domain in each study were sought (e.g. for all measures, time points, analyses), and if not, the methods used to decide which results to collect. | Page 4-5 |
|  | 10b | List and define all other variables for which data were sought (e.g. participant and intervention characteristics, funding sources). Describe any assumptions made about any missing or unclear information. | Page 4-5 |
| Study risk of bias assessment | 11 | Specify the methods used to assess risk of bias in the included studies, including details of the tool(s) used, how many reviewers assessed each study and whether they worked independently, and if applicable, details of automation tools used in the process. | Page 5 |
| Effect measures | 12 | Specify for each outcome the effect measure(s) (e.g. risk ratio, mean difference) used in the synthesis or presentation of results. | Page 5 |
| Synthesis methods | 13a | Describe the processes used to decide which studies were eligible for each synthesis (e.g. tabulating the study intervention characteristics and comparing against the planned groups for each synthesis (item #5)). | Page 4 |
|  | 13b | Describe any methods required to prepare the data for presentation or synthesis, such as handling of missing summary statistics, or data conversions. | Page 4-5 |
|  | 13c | Describe any methods used to tabulate or visually display results of individual studies and syntheses. | Page 5 |
|  | 13d | Describe any methods used to synthesize results and provide a rationale for the choice(s). If meta-analysis was performed, describe the model(s), method(s) to identify the presence and extent of statistical heterogeneity, and software package(s) used. | Page 5 |
|  | 13e | Describe any methods used to explore possible causes of heterogeneity among study results (e.g. subgroup analysis, meta-regression). | Page 5 |
|  | 13f | Describe any sensitivity analyses conducted to assess robustness of the synthesized results. | N/A |
| Reporting bias assessment | 14 | Describe any methods used to assess risk of bias due to missing results in a synthesis (arising from reporting biases). | Page 5 |
| Certainty assessment | 15 | Describe any methods used to assess certainty (or confidence) in the body of evidence for an outcome. | N/A |
| **RESULTS** | | |  |
| Study selection | 16a | Describe the results of the search and selection process, from the number of records identified in the search to the number of studies included in the review, ideally using a flow diagram. | Figure 1 |
|  | 16b | Cite studies that might appear to meet the inclusion criteria, but which were excluded, and explain why they were excluded. | Page 5–6 |
| Study characteristics | 17 | Cite each included study and present its characteristics. | Page 5-7 |
| Risk of bias in studies | 18 | Present assessments of risk of bias for each included study. | Table S2 |
| Results of individual studies | 19 | For all outcomes, present, for each study: (a) summary statistics for each group (where appropriate) and (b) an effect estimate and its precision (e.g. confidence/credible interval), ideally using structured tables or plots. | Figure 2, 3 |
| Results of syntheses | 20a | For each synthesis, briefly summarise the characteristics and risk of bias among contributing studies. | Table 1, S2 |
|  | 20b | Present results of all statistical syntheses conducted. If meta-analysis was done, present for each the summary estimate and its precision (e.g. confidence/credible interval) and measures of statistical heterogeneity. If comparing groups, describe the direction of the effect. | Page 7,  Figure 2, 3 |
|  | 20c | Present results of all investigations of possible causes of heterogeneity among study results. | Page 7 |
|  | 20d | Present results of all sensitivity analyses conducted to assess the robustness of the synthesized results. | N/A |
| Reporting biases | 21 | Present assessments of risk of bias due to missing results (arising from reporting biases) for each synthesis assessed. | N/A |
| Certainty of evidence | 22 | Present assessments of certainty (or confidence) in the body of evidence for each outcome assessed. | N/A |
| **DISCUSSION** | | |  |
| Discussion | 23a | Provide a general interpretation of the results in the context of other evidence. | Page 8-10 |
|  | 23b | Discuss any limitations of the evidence included in the review. | Page 10 |
|  | 23c | Discuss any limitations of the review processes used. | Page 10 |
|  | 23d | Discuss implications of the results for practice, policy, and future research. | Page 10 |
| **OTHER INFORMATION** | | |  |
| Registration and protocol | 24a | Provide registration information for the review, including register name and registration number, or state that the review was not registered. | N/A |
|  | 24b | Indicate where the review protocol can be accessed, or state that a protocol was not prepared. | N/A |
|  | 24c | Describe and explain any amendments to information provided at registration or in the protocol. | N/A |
| Support | 25 | Describe sources of financial or non-financial support for the review, and the role of the funders or sponsors in the review. | N/A |
| Competing interests | 26 | Declare any competing interests of review authors. | Page 11 |
| Availability of data, code and other materials | 27 | Report which of the following are publicly available and where they can be found: template data collection forms; data extracted from included studies; data used for all analyses; analytic code; any other materials used in the review. | N/A |

PRISMA, Preferred Reporting Items for Systematic Reviews and Meta-Analyses; N/A, not applicable.

**PICO protocol**

The PICO question was, “Is there a change in muscular strength after bariatric metabolic surgery?”.

1. Population: Patients who underwent bariatric metabolic surgery
2. Intervention: Bariatric metabolic surgery
3. Comparison: Baseline muscle strength
4. Outcomes: Change in muscle strength

Eligible articles were longitudinal studies conducted on patients of all ages and sexes who underwent bariatric metabolic surgery. The studies reported changes in handgrip strength pre- and postoperatively and were published in English without any publication year restrictions.
